# Role of Hypoxia-Inducible Factors in Respiratory Syncytial Virus Infection-Associated Lung Disease

**DOI:** 10.3390/ijms26073182

**Published:** 2025-03-29

**Authors:** Dorothea R. Morris, Yue Qu, Aline Haas de Mello, Yava L. Jones-Hall, Tianshuang Liu, Meredith Weglarz, Teodora Ivanciuc, Roberto P. Garofalo, Antonella Casola

**Affiliations:** 1 Department of Microbiology & Immunology, The University of Texas Medical Branch, Galveston, TX 77555, USA; dormorri@utmb.edu (D.R.M.); meweglar@utmb.edu (M.W.); rpgarofa@utmb.edu (R.P.G.); 2 School of Population & Public Health, The University of Texas Medical Branch, Galveston, TX 77555, USA; 3 Department of Pediatrics, The University of Texas Medical Branch, Galveston, TX 77555, USA; yuqu@utmb.edu (Y.Q.); alhaasde@utmb.edu (A.H.d.M.); tiliu@utmb.edu (T.L.); teivanci@utmb.edu (T.I.); 4 School of Veterinary Medicine & Biomedical Science, Texas A&M University, College Station, TX 77843, USA; yavajh@cvm.tamu.edu

**Keywords:** respiratory syncytial virus, HIF-1a, HIF-2a, PX478, PT2385

## Abstract

Hypoxia-inducible factors (HIFs) are transcription factors that enable cells to adapt to low-oxygen environments. Viruses can exploit this pathway to enhance infection, making HIF modulation a potential antiviral strategy. In previous in vitro studies, we found that respiratory syncytial virus (RSV) stabilizes HIFs under normoxic conditions with inhibition of HIF-1α reducing replication. Despite several HIF-modulating compounds being tested or approved in other non-infectious models, little is known about their efficacy against respiratory viruses in relevant animal models. This study aimed to characterize the disease-modulating properties and antiviral potential of HIF-1α (PX478) and HIF-2α PT2385 inhibitors in RSV-infected BALB/c mice. We found that the inhibition of HIF-1α worsened clinical disease parameters while simultaneously improving airway function. Blocking HIF-1α also significantly reduced peak RSV replication in the lung. In contrast, the inhibition of HIF-2α was associated with improved clinical parameters, no changes in airway function, and reduced viral replication following RSV infection. The analysis of lung cells found significant modification in the T-cell compartment that correlated with changes in lung pathology and viral titers for each HIF inhibitor. This study underscores the differential roles of HIF proteins in RSV infection and highlights the need for further characterization of compounds currently in use or under therapeutic consideration.

## 1. Introduction

Respiratory syncytial virus (RSV) is a prominent cause of respiratory infections among infants, young children, and the elderly. Each year, there are an estimated 33 million infections and 150,000 deaths [1]. Infection with RSV often results in damage to the airways and a dysregulated immune response, contributing to the development of bronchiolitis and pneumonia. Despite the availability of prophylactic treatment and vaccines licensed for use in pregnant women and elderly patients, there are no therapeutic agents licensed for the treatment of RSV infection [2,3,4]. Furthering our understanding of the pathology of RSV is important to protecting these vulnerable populations.

As an obligate intracellular pathogen, viruses rely on host-cell machinery to establish productive infections. Several large DNA viruses and some RNA viruses with complex replication cycles have been shown to modify cellular metabolism to achieve virion assembly by increasing sources of energy and generating pools of free nucleotides, amino acids, lipid materials, and glycoproteins [5]. One mechanism frequently associated with this is the manipulation of the hypoxia-inducible factor (HIF) pathway. As the gatekeeper to a diverse biological network, the utilization of the HIF pathway enables many viruses to create a cellular environment conducive to viral replication, persistence, and immune evasion. The manipulation of this pathway has been observed with several DNA and RNA viruses, even under oxygen-rich conditions [6,7]. The significance of HIF in cancer pathology has led much of the existing literature to focus on viruses known to induce tumor formation as part of their pathogenic mechanism [6,7]. Surprisingly, limited research has explored the clinical manifestations and pathological contributions of HIFs to the pathophysiology of viral respiratory infections [6,7,8,9].

HIFs are transcription factors that regulate the expression of a wide range of target genes in response to low oxygen levels [10]. These target genes have crucial roles in diverse biological processes, enabling cells to adapt and survive under hypoxic conditions. The HIF complex is a heterodimer consisting of an inducible a subunit (HIF-1α, HIF-2α, or HIF-3α) and a constitutively expressed β subunit (HIF-1β) [11]. While there is overlap in their functionality, the HIF-α isoforms also exhibit distinct transcriptional regulation in response to hypoxia and disease states [12,13,14]. HIF-1α is the most extensively studied isoform of the HIF family. Expressed in nearly all cell types, HIF-1α is the master regulator of cellular and systemic responses to hypoxia. Genes activated by HIF-1α modify a broad range of processes, including energy metabolism, angiogenesis, epithelial–mesenchymal transition (EMT), and immune cell regulation [12,14]. HIF-2α, though not as thoroughly described as HIF-1, has been shown to exhibit a more limited distribution; it is predominantly expressed in highly vascularized organs such as the kidneys, liver, and lungs. Genes upregulated by HIF-2α in models of cancer and non-infectious tissue repair are primarily involved in erythropoiesis, vascular development, and iron metabolism [14]. HIF-3α is the most recent of the HIF isoforms to be identified. Because of this, the precise distribution and function of HIF-3α are still being investigated.

Our laboratory recently found that RSV stabilizes both HIF-1α and HIF-2α in primary small airway epithelial cells and diverts core metabolic activity towards the glycolytic pathway in an HIF-1α-dependent manner [15]. The inhibition of HIF-1α, but not HIF-2α, was associated with reduced viral replication. Given the multiple roles of HIFs in both the epithelial and immune compartments, we investigated their involvement in RSV lung disease and pathology by utilizing established inhibitors of HIF-1a (PX478) and HIF-2α (PT2385) in a BALB/c mouse model. Our results underscore the distinct functionality of the individual HIF-α subunits in the development of RSV pathogenesis and indicate the importance of testing HIF therapeutics approved for conditions such as cancer in complex viral respiratory infection biological models for potential benefits but also unwanted side effects.

## 2. Results

### 2.1. Effect of PX478 Administration on RSV Infection-Associated Disease Parameters and Viral Replication

To investigate the role of HIF-1α in RSV disease, groups of BALB/c mice were initially administered PX478 at a dose of 20 mg/kg by oral gavage every 24 h (PXQD) over the 10-day infection period (Figure S1A). Shortly after the first dose of PX478, mice were anesthetized and intranasally (IN) infected with RSV at a dose of 5 × 10^6^ PFU. We found that PXQD treatment was associated with worsening body weight and illness score, characterized by an inability to regain weight regardless of infection status (Figure S1B,C).

To assess airway function during RSV infection, we measured bronchoconstriction using whole-body plethysmography, as previously described [16]. Uninfected mice that received PX478 showed no significant alterations of Penh values at any timepoint tested compared to the PBS/PBS control group. RSV-infected PX478-treated mice showed significant improvement in Penh at days 1, 5, and 6 p.i., with values comparable to the PBS/PBS control mice throughout the disease course (Figure S1D).

Next, we measured the total protein level in the BAL fluid as an indicator of epithelial damage. PX478 treatment did not alter BAL protein levels in uninfected mice, while RSV-infected PX478-treated mice showed significant improvements in total protein at day 4 p.i. compared to the RSV/PBS control mice (Figure S1E).

To assess the effect of PX478 treatment on viral replication, we performed plaque assays at the early and peak timepoints of day 2 and 4 p.i. We found that PX478 treatment significantly reduced replication on days 2 and 4 p.i. when compared to the RSV/PBS control mice. However, RSV replication in RSV PX478-treated mice persisted through day 7 p.i., a timepoint when RSV/PBS control mice had achieved viral clearance, with some mice having detectable replication in the lung tissue as late as day 10 p.i. (Figure S1F).

**Figure 1:**
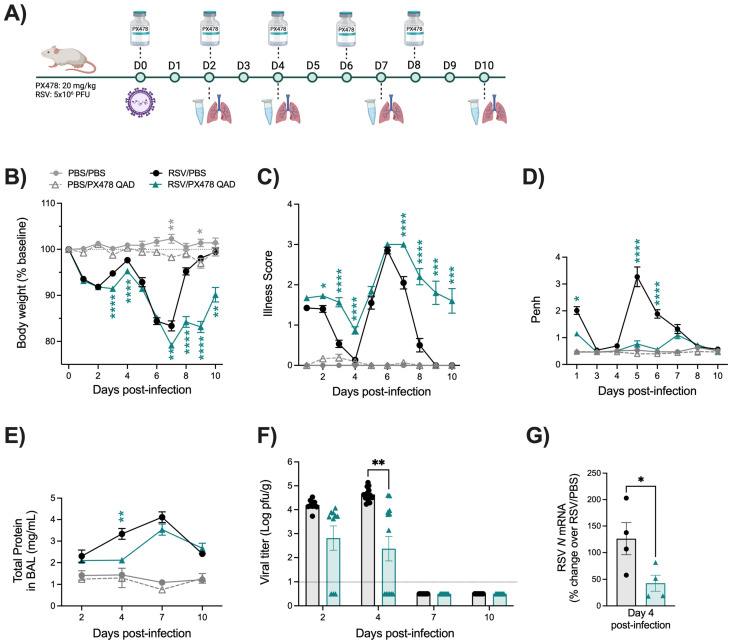
Assessment of clinical disease, airway function, and viral replication following HIF-1α inhibition during RSV infection. The experimental design for mice treated with PX478 QAD is described in (**A**). Following treatment, all mice were monitored daily for changes in (**B**) body weight and (**C**) illness score. (**D**) Bronchoconstriction, represented by baseline Penh, was measured by plethysmography. At days 2, 4, 7, and 10 p.i., (**E**) total protein was measured in the BAL fluid, and the lung was collected for assessment of (**F**) viral replication by plaque assay. At day 4 p.i., (**G**) RSV N mRNA levels were measured by RT-qPCR. For clinical disease, data are pooled from four independent experiments (PBS/PBS n = 6–8, PBS/PX n = 6–18, RSV/PBS n = 10–30, RSV/PX n = 9–30 mice/group). For bronchoconstriction, data are pooled from three independent experiments (PBS/PBS and PBS/PX n = 6–12, RSV/PBS and RSV/PX n = 6–30 mice/group). For total protein, data are pooled from two independent experiments (PBS/PBS and PBS/PX n = 4–6, RSV/PBS and RSV/PX n = 8–10 mice/group). For viral replication by plaque assay, data are pooled from two independent experiments (n = 10–14 mice/group). For RT-qPCR, data are from one independent experiment (n = 4 mice/group). Data are expressed as mean ± SEM. Significant results were determined by two-way mixed ANOVA (**B**–**F**) and unpaired *t*-test (**G**) (* *p* ≤ 0.05, ** *p* ≤ 0.01, *** *p* ≤ 0.001, **** *p* ≤ 0.0001).

To improve the toxicity observed on body weight loss and illness in the PBS/PXQD-treated mice, we tested lower doses of the compound (5 to 15 mg/kg) for changes in body weight and viral replication. Following the same treatment schedule, we found body weight to be improved for some of the doses in PBS mice treated with PX478, but none of the RSV mice treated with PX478 had comparable antiviral activity. The administration of PX478 at 20 mg/kg was then changed to every 48 h (PXQAD) over the 10-day infection period (Figure 1A). Uninfected mice that received the compound on alternate days no longer exhibited substantial weight loss or heightened illness in comparison to the PBS/PBS control mice. However, RSV-infected mice treated with PXQAD continued to demonstrate significantly worse body weight loss and illness scores in comparison to the RSV/PBS mice, although these mice showed signs of recovery at the later stages of the disease course (Figure 1B,C).

Lung function was assessed by measuring bronchoconstriction (Penh) and BAL total protein levels. RSV-infected mice that received PXQAD had significant improvements in Penh at days 1, 5, and 6 p.i., with values comparable to the PBS/PBS control mice throughout the disease course (Figure 1D). RSV/PXQAD mice also had significant improvement in BAL total protein level at day 4 p.i. compared to the RSV/PBS control mice (Figure 1E).

To assess the effect of PXQAD treatment on viral replication, we performed plaque assays at early and peak timepoints of day 2 and 4 p.i., as well as days 7 and 10 p.i., to assess viral clearance. RSV/PXQAD mice showed significantly reduced RSV replication at day 4 p.i., with no significant difference in viral clearance at days 7 and 10 p.i. when compared to the RSV/PBS group (Figure 1F). The change in peak RSV replication was further supported by reductions in the RSV *N* gene by RT-qPCR at day 4 p.i. (Figure 1G). All subsequent experiments described below were then performed using the PXQAD treatment protocol.

### 2.2. Effect of PX478 Administration on RSV-Induced HIF-1a Target Genes

**Figure 2:**
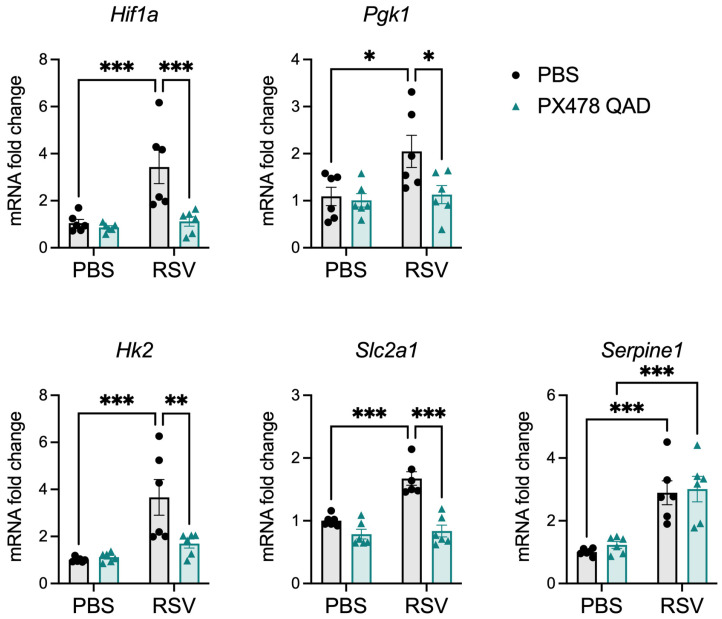
Effect of PX478 on Hif1a (HIF-1α) and HIF-associated target genes in the lungs of mice during RSV infection. Mice were euthanized at day 2 p.i., and RNA was extracted from the lung tissue. The gene expression of Hif1a; the HIF-1α targets Pgk1, Hk2, and Slc2a1 (GLUT1); and the HIF-2α target Serpine1 (PAI-1) was measured by RT-qPCR (n = 6 mice/group). Data are expressed as mean ± SEM. Significant results were determined by two-way ANOVA followed by Bonferroni’s multiple comparisons test (* *p* ≤ 0.05, ** *p* ≤ 0.01, *** *p* ≤ 0.001).

To confirm inhibition of RSV-induced HIF-1α activation by PX478 treatment, we analyzed the expression of Hif1a and the HIF-1α target genes phosphoglycerate kinase 1 (Pgk1), hexokinase 2 (Hk2), and solute carrier family 2 (facilitated glucose transporter) member 1 (Slc2a1) at day 2 p.i. in lungs of mice untreated or treated with the compound (Figure 2). RSV infection resulted in the upregulation of HIF-1α and HIF-1α target genes, which was significantly reduced by PX478 treatment. The specificity of PX478 was determined by assessment of the HIF-2α-dependent genes erythropoietin (Epo) and Serpine1. Epo was not detectable in the lung tissue of any mice. RSV infection induced the expression of Serpine1, which was not changed by PX478 treatment, indicating the specificity of the compound.

### 2.3. Inhibition of HIF-1a Leads to Reduced Immune Responses Following RSV Infection

**Figure 3:**
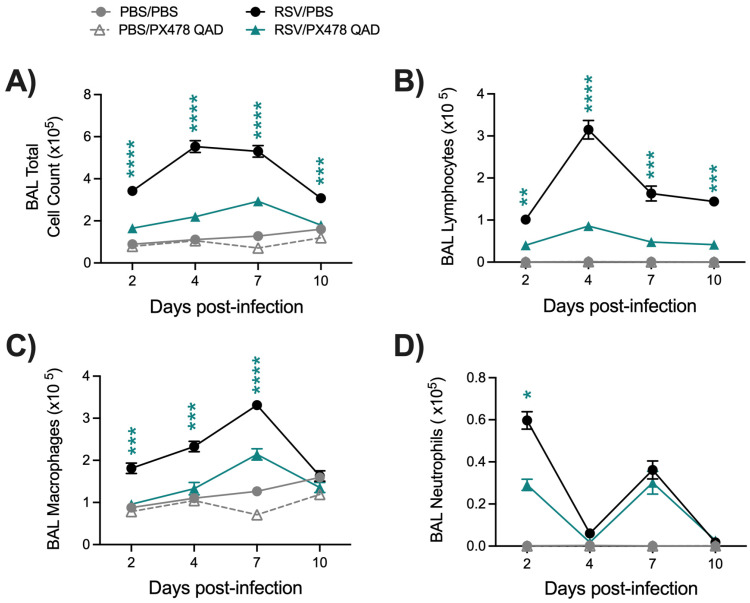
Differential cell count of the BAL fluid following HIF-1α inhibition during RSV infection. At days 2, 4, 7, or 10 p.i., BAL fluid was collected from mice that had been treated or not with PX478 QAD. This BAL was used to obtain (**A**) total cell counts, as well as differential cell counts consisting of (**B**) lymphocytes, (**C**) macrophages, and (**D**) neutrophils. Data are pooled from two independent experiments (PBS/PBS and PBS/PX478 n = 4–6, RSV/PBS and RSV/PX478 n = 10 mice/group). Data are expressed as mean ± SEM. Significant results were determined by two-way mixed ANOVA (* *p* ≤ 0.05, ** *p* ≤ 0.01, *** *p* ≤ 0.001, **** *p* ≤ 0.0001).

The immune response to an RSV infection plays a key role in determining disease severity and the control of RSV replication [17]. To understand the impact of HIF-1α inhibition on the immune response to RSV, we first evaluated the cellular composition of the BAL fluid. Uninfected mice that received PXQAD showed no significant changes in BAL cellular composition as compared to the PBS/PBS control mice (Figure 3A–D). On the other hand, RSV-infected mice that received the compound demonstrated significant reductions in the number of total cells present in the BAL (Figure 3A), specifically in the number of lymphocytes (Figure 3B) and macrophages (Figure 3C) at all timepoints tested compared to RSV/PBS mice. Neutrophil cell counts were also significantly reduced at day 2 p.i. but remained similar to the RSV/PBS control mice at all other timepoints (Figure 3D). Together, these data suggest HIF-1α is important for the recruitment of immune cells within the airway space.

**Figure 4:**
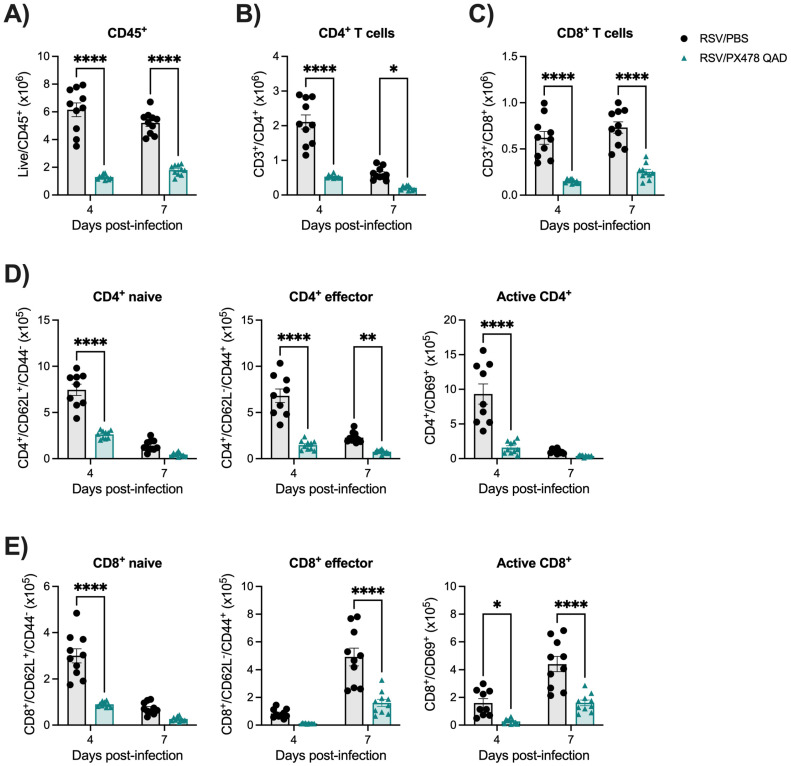
Assessment of CD4^+^ and CD8^+^ T cells following HIF-1α inhibition during RSV infection. Whole lung tissue was collected at peak RSV replication and a timepoint of viral clearance, days 4 and 7 p.i., respectively. A single-cell suspension was prepared, stained with live/dead cell dye and fluorochrome-conjugated antibodies, and acquired by flow cytometry. Data were analyzed following the gating strategy in Figure S2. Quantification of absolute cell counts of (**A**) Live CD45+, (**B**) CD4^+^ T cells, (**C**) CD8^+^ T cells, (**D**) CD4^+^ naive, effector, and active cells, and (**E**) CD8^+^ naive, effector, and active cells are shown. Data are pooled from two independent experiments (n = 9–10 mice/group). Data are expressed as mean ± SEM. Significant results were determined by two-way mixed ANOVA (* *p* ≤ 0.05, ** *p* ≤ 0.01, **** *p* ≤ 0.0001).

CD4^+^ and CD8^+^ T cells play a fundamental role in controlling RSV replication and clearance within the host [17,18,19]. With reduced lymphocyte numbers in the BAL and the complex outcome of viral replication (prolonged viral replication in the PXQD treatment schedule), we next wanted to assess the impact of HIF-1α inhibition on the T-cell population within the lung tissue. To do so, flow cytometry analysis was performed using whole lung tissue at days 4 and 7 p.i., as depicted in Figure S2. RSV-infected mice treated with PXQAD demonstrated a significant reduction in total leukocytes (Figure 4A) and CD4^+^ (Figure 4B) and CD8^+^ (Figure 4C) T cells present in the lungs at days 4 and 7 p.i. compared to the RSV/PBS control mice. We then further examined CD4^+^ T-cell subpopulations, including naïve, effector, and active cells. We found the absolute cell numbers for all three CD4^+^ subpopulations to be significantly reduced at day 4 p.i. (Figure 4D and Table S1). In accordance, the percentage of effector and active CD4^+^ cells decreased, while the percent of naïve CD4^+^ cells increased as compared to the RSV/PBS control mice (Table S1). At day 7 p.i., the absolute cell counts of effector CD4^+^ T cells remained significantly reduced in the lung tissue of RSV/PXQAD mice. The absolute cell counts for naïve and active CD4^+^ T cells and the percent change for all three CD4^+^ subpopulations were comparable to the RSV/PBS control mice at day 7 p.i. (Figure 4D and Table S1).

We similarly assessed CD8^+^ subpopulations, including naïve, effector, and active cells. At day 4 p.i., RSV/PXQAD mice had a trend for reduced absolute cell number of effector cells and significant reductions in the absolute cell numbers for the naïve and active CD8^+^ subpopulations as compared to the RSV/PBS control mice (Figure 4E and Table S1). In accordance, the percentage of effector and active CD8^+^ cells decreased, while the percent of naïve CD8^+^ cells increased (Table S1). At day 7 p.i., the absolute cell counts of effector and active CD8^+^ T cells were significantly reduced (Figure 4E). Similarly, the percent change of effector CD8^+^ cells was reduced, while the percent of naïve and active CD8^+^ T cells remained comparable to the RSV/PBS control mice (Table S1). Collectively, these data indicate that HIF-1α plays an important role in regulating T-cell responses during RSV infection by modulating T-cell recruitment to the lung and their activation.

**Figure 5:**
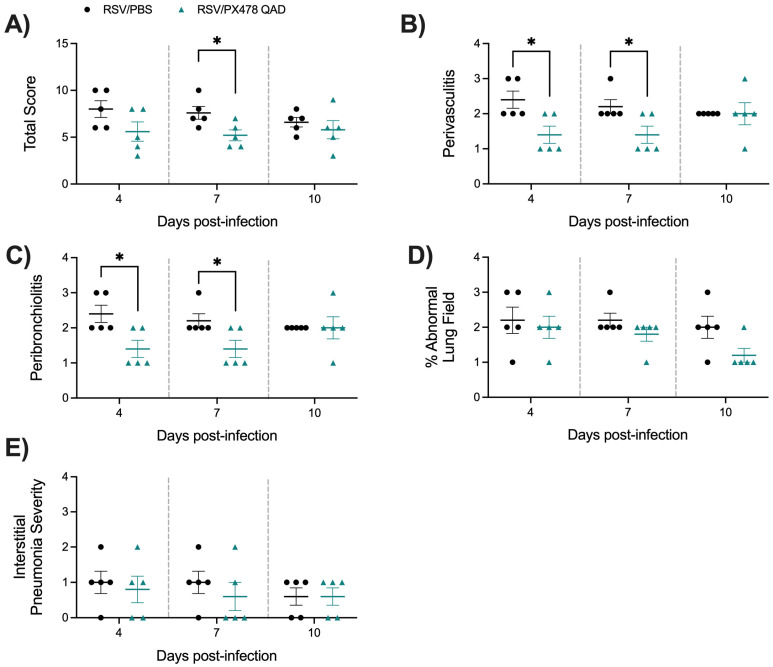
Histopathological scoring of lung tissue following HIF-1α inhibition during RSV infection. At days 4, 7, and 10 p.i., the left lung was collected from RSV-infected mice that had received PBS or PX478 QAD and subjected to FFPE. Cuts of lung tissue were stained with H&E and observed under a microscope at 10× magnification. The (**A**) total score was calculated, consisting of scores for (**B**) perivasculitis, (**C**) peribronchiolitis, (**D**) percent abnormal lung field, and (**E**) interstitial pneumonia. Data are representative of one independent experiment (n = 5 mice/group). Data are expressed as mean ± SEM. Significant results were determined by an unpaired Student’s *t*-test (* *p* ≤ 0.05).

**Figure 6:**
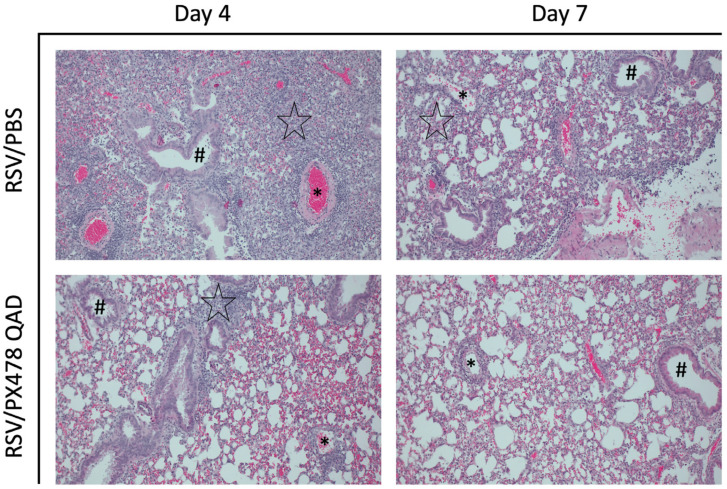
Histopathological imaging of lung tissue following HIF-1α inhibition during RSV infection. At days 4 and 7 p.i., the left lung was collected from mice that had received PBS or PX478 every 48 h and subjected to FFPE. Cuts of lung tissue were stained with H&E and observed under a microscope at 10× magnification. Perivasculitis is indicated by an asterisk. Peribronchiolitis is indicated by the hash sign. Interstitial pneumonia is indicated by the black star. No interstitial pneumonia was present in RSV/PX478 QAD on day 7. Representative images are shown here (n = 5 mice/group).

Last, we assessed the lung tissue of mice that received PXQAD by histopathology. Based on the scoring determined by a board-certified veterinarian with expertise in mouse lung morphology, uninfected mice that received PXQAD remained comparable to the PBS/PBS control mice in all categories and at all timepoints tested. RSV/PXQAD mice demonstrated significant improvements in the total lung score as compared to the RSV/PBS control mice (Figure 5A). Specifically, perivasculitis (Figure 5B) and peribronchiolitis (Figure 5C) were significantly improved at days 4 and 7 p.i. compared to the RSV/PBS control mice. This finding is consistent with the reduction in immune cells noted in the BAL and lung tissue of these mice. No significant differences in the percent of abnormal lung field (Figure 5D) or the severity of interstitial pneumonia (Figure 5E) were appreciated at any timepoint. Representative images of the lung tissue for RSV/PBS and RSV/PXQAD mice are shown in Figure 6. Collectively, these data suggest HIF-1α contributes to the worsening of airway function and tissue damage during the acute phase of RSV infection.

### 2.4. Effect of PT2385 Administration on RSV Infection-Associated Disease Parameters and Viral Replication

**Figure 7:**
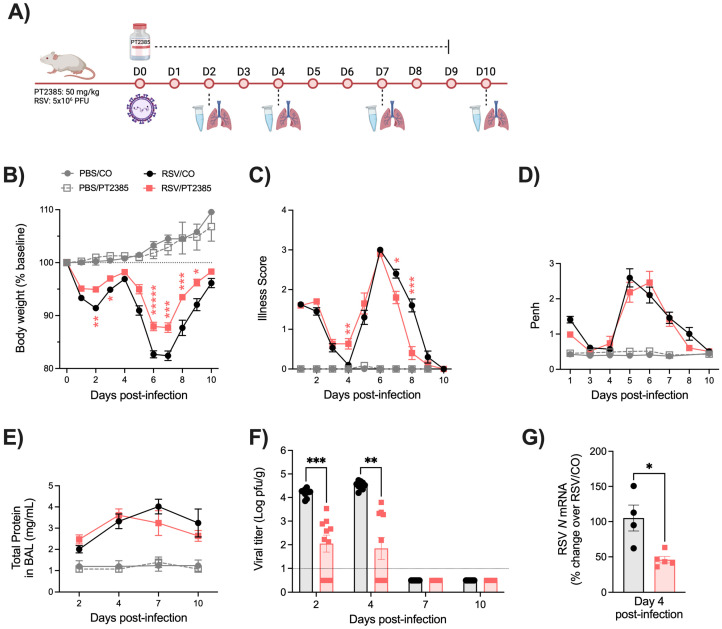
Assessment of clinical disease, airway function, and viral replication following HIF-2α inhibition during RSV infection. The experimental design for mice treated with PT2385 is described in (**A**). Following treatment, all mice were monitored daily for changes in (**B**) body weight and (**C**) illness score. (**D**) Bronchoconstriction, represented by baseline Penh, was measured by plethysmography. At days 2, 4, 7, and 10 p.i., (**E**) total protein was measured in the BAL fluid, and the right lung was collected for assessment of (**F**) viral replication by plaque assay. At day 4 p.i., (**G**) RSV N mRNA levels were measured by RT-qPCR. For clinical disease, data are pooled from four independent experiments (PBS/CO n = 6–12, PBS/PT n = 6–18, RSV/CO n = 6–24, RSV/PT n = 6–24 mice/group). For bronchoconstriction, data are pooled from three independent experiments (PBS/CO and PBS/PT n = 6–12, RSV/CO and RSV/PT n = 6–24 mice/group). For total protein, data are pooled from two independent experiments (PBS/CO and PBS/PT n = 4–5, RSV/CO and RSV/PT n = 5–10 mice/group). For viral replication by plaque assay, data are pooled from two independent experiments (n = 9–10 mice/group). For RT-qPCR, data are from one independent experiment (n = 4–5 mice/group). Data are expressed as mean ± SEM. Significant results were determined by two-way mixed ANOVA (**B**–**F**) and unpaired *t*-test (**G**) (* *p* ≤ 0.05, ** *p* ≤ 0.01, *** *p* ≤ 0.001, ***** *p* ≤ 0.00001).

While there is a degree of functional overlap between the HIF-α subunits, they have also been demonstrated to govern a distinct range of biological activities [14]. To better understand the specific role of HIF-2α during RSV infection, mice were administered PT2385 by oral gavage every 24 h at a dose of 50 mg/kg (Figure 7A). Shortly after the first dose, mice were anesthetized and infected with RSV at a dose of 5 × 10^6^ PFU. Uninfected mice that received PT2385 were found to have similar body weight loss and illness scores as the PBS/CO control mice (Figure 7B,C). RSV-infected mice that received PT2385 demonstrated significant improvements in body weight loss at several timepoints over the 10-day disease course as compared to the RSV/CO control mice (Figure 7B). Interestingly, the illness score in RSV/PT2385 mice was worse at day 4 p.i., but treated animals showed a quicker recovery at later timepoints of infection when compared to the RSV/CO control mice (Figure 7C).

To assess airway function during RSV infection, we measured bronchoconstriction and BAL total protein levels. Uninfected mice that received PT2385 showed no significant alterations to Penh values at any timepoint tested as compared to the PBS/CO control mice. Similarly, RSV-infected mice treated with PT2385 had Penh values similar to those of the RSV/CO control mice throughout the 10-day disease course (Fig. 7D). We observed no significant differences in total protein for all mice tested as compared to their respective control groups (Figure 7E).

The assessment of viral replication in the lung tissue, assessed by plaque assay and viral gene expression, showed RSV/PT2385 mice to have reduced RSV replication at early and peak timepoints of days 2 and 4 p.i. (Figure 7F,G). By days 7 and 10 p.i., no replicating virus was detected in RSV/PT2385 mice, indicating successful clearance of RSV from the lung tissue. Collectively, these data suggest HIF-2a contributes to RSV-induced disease and control of viral replication.

### 2.5. Effect of PT2385 Administration on RSV-Induced HIF-2a Target Genes

**Figure 8:**
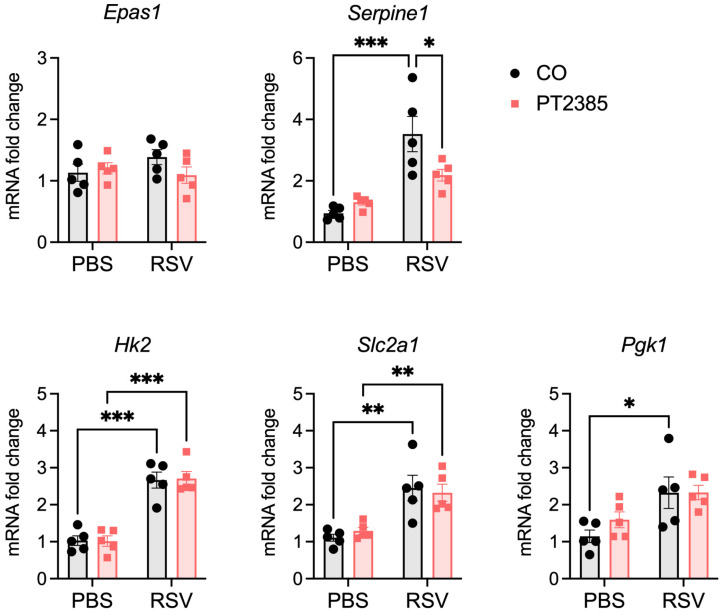
Effect of PT2385 on Epas1 (HIF-2α) and HIF-associated target genes in lungs of mice during RSV infection. A group of mice were euthanized at day 2 p.i., lungs were harvested, and total lung RNA was extracted. Gene expression of Epas1 (HIF-2α), the HIF-2α target Serpine1 (PAI-1), and the HIF-1α targets Pgk1, Slc2a1 (GLUT1), and Hk2 was measured by RT-qPCR (n = 5 mice/group). Data are expressed as mean ± SEM. Significant results were determined by two-way ANOVA followed by Bonferroni’s multiple comparisons test (* *p* ≤ 0.05, ** *p* ≤ 0.01, *** *p* ≤ 0.001).

To confirm inhibition of RSV-induced HIF-2α activation by PT2385 treatment, we analyzed the expression of Epas1 (HIF-2α) and the HIF-2α target genes Serpine1 and Epo at day 2 p.i. in lungs of mice untreated or treated with the compound (Figure 8). There was no change in baseline HIF-2α gene expression (Epas1) following RSV infection, which is different from what we observed for HIF-1α. We confirmed the induction of the HIF-2α target gene Serpine1, which was significantly reduced by PT2385 treatment. Epo was not detectable in the lung tissue of any mice. There was no change in the RSV-induced expression of HIF-1α-dependent genes Hk2, Slc2a1, and Pgk1 following treatment, indicating the specificity of the compound (Figure 8).

### 2.6. Inhibition of HIF-2a Leads to Increased Immune Activity in the BAL and Lung Tissue of RSV-Infected Mice

**Figure 9:**
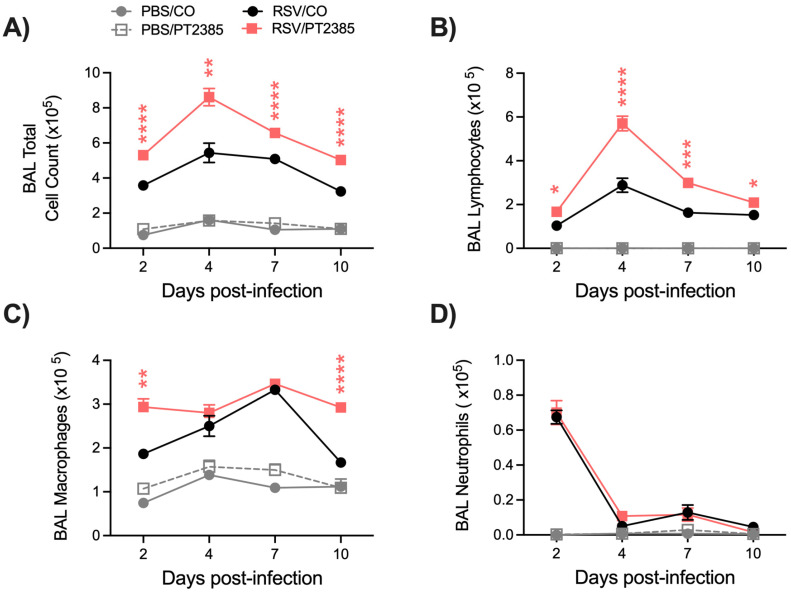
Differential cell count of the BAL fluid following HIF-2α inhibition during RSV infection. At days 2, 4, 7, and 10 p.i., BAL fluid was collected and used to obtain (**A**) total cell counts, as well as differential cell counts consisting of (**B**) lymphocytes, (**C**) macrophages, and (**D**) neutrophils. Data are pooled from two independent experiments (PBS/CO and PBS/PT n = 4–6, RSV/CO and RSV/PT n = 10–15 mice/group). Data are expressed as mean ± SEM. Significant results were determined by mixed-effects analysis (* *p* ≤ 0.05, ** *p* ≤ 0.01, *** *p* ≤ 0.001, **** *p* ≤ 0.0001).

We had previously demonstrated HIF-2α to be dispensable for the control of RSV replication in human small airway epithelial cells. Given the antiviral outcome in RSV/PT2385 mice, we next wanted to understand the contribution of HIF-2α to the immune response during an RSV infection. We first evaluated the cellular composition of the BAL fluid. Uninfected mice that received PT2385 showed no significant difference in BAL cellular composition compared to the PBS/CO control group (Figure 9A–D). RSV-infected mice that received PT2385 demonstrated a significant increase in the total number of cells present in the BAL throughout the course of the infection (Figure 9A), particularly for the number of lymphocytes (Figure 9B), compared to the PBS/CO control group. Macrophage cell counts were also significantly increased at days 2 and 10 p.i. (Figure 9C), while neutrophil cell counts remained comparable to the RSV/CO control mice (Figure 9D).

**Figure 10:**
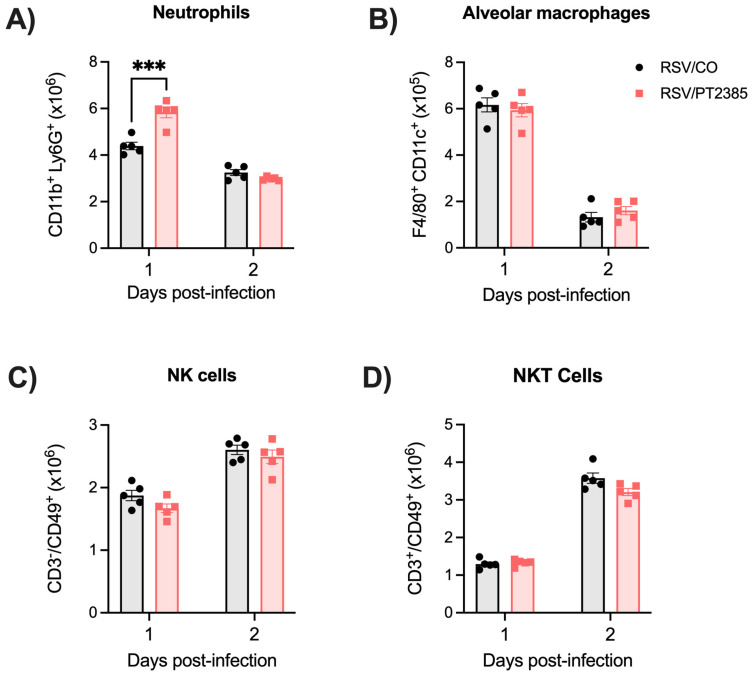
Assessment of neutrophils, alveolar macrophages, natural killer cells, and NKT cells following HIF-2α inhibition during RSV infection. Mice were euthanized at days 1 and 2 p.i., and whole lung tissue was collected. A single-cell suspension was prepared, stained with live/dead cell dye and fluorochrome-conjugated antibodies, and acquired by flow cytometry. Data were analyzed following the gating strategy in Figure S3. Absolute cell counts for (**A**) neutrophils, (**B**) alveolar macrophages, (**C**) NK cells, and (**D**) NKT cells are shown here. Data are expressed as mean ± SEM (n = 5 mice/group). Significant results were determined by two-way ANOVA (*** *p* ≤ 0.001).

Given the increase in some innate cells in the BAL, we next assessed a few innate immune cells speculated to contribute to RSV antiviral activity by flow cytometry. Whole lung tissue was collected from RSV-infected mice treated or not with PT2385 at days 1 and 2 p.i., the peak of innate immune cell activity in RSV-infected mice. We assessed the tissue for alveolar macrophages, neutrophils, natural killer (NK) cells, and natural killer T (NKT) cells. We found a significant increase in the absolute cell count of neutrophils on day 2 p.i. in the lungs of RSV/PT2385 mice as compared to the RSV/CO control mice (Figure 10A). Absolute cell counts of alveolar macrophages, NK cells, and NKT cells were comparable between the two groups (Figure 10B–D).

**Figure 11:**
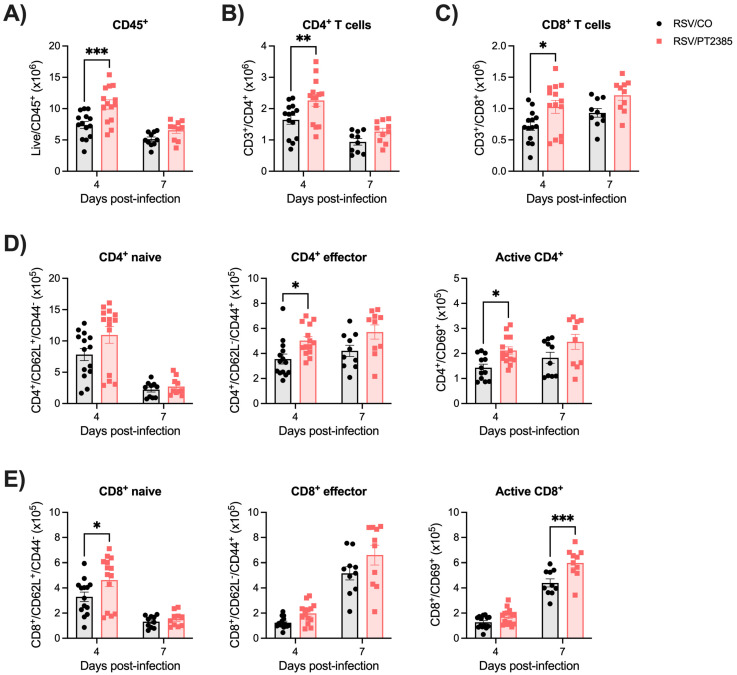
Assessment of CD4^+^ and CD8^+^ T cells following HIF-2α inhibition during RSV infection. Whole lung tissue was collected at peak RSV replication and a timepoint of viral clearance, days 4 and 7 p.i., respectively. A single-cell suspension was prepared, stained with live/dead cell dye and fluorochrome-conjugated antibodies, and acquired by flow cytometry. Data were analyzed following the gating strategy in Figure S2. Quantification of absolute cell counts of (**A**) Live CD45+, (**B**) CD4^+^ T cells, (**C**) CD8^+^ T cells, (**D**) CD4^+^ naive, effector, and active cells, and (**E**) CD8^+^ naive, effector, and active cells are shown. Data are pooled from two to three independent experiments (n = 10–14 mice/group). Data are expressed as mean ± SEM. Significant results were determined by a two-way mixed ANOVA (* *p* ≤ 0.05, ** *p* ≤ 0.01, *** *p* ≤ 0.001).

As with HIF-1α, we next assessed CD4^+^ and CD8^+^ T-cell responses by flow cytometry analysis. RSV-infected mice treated with PT2385 demonstrated a significant increase in total leukocytes (Figure 11A) and CD4^+^ (Figure 11B) and CD8^+^ (Figure 11C) T cells in the lung at day 4 p.i. We further analyzed the CD4^+^ T-cell subpopulations of naïve, effector, and active T cells. In RSV/PT2385 mice at day 4 p.i., we observed an increasing trend in the total number of CD4^+^ naïve cells as well as a significant increase in the absolute number of CD4^+^ effector and active T cells as compared to the RSV/CO control mice (Figure 11D). The percent change at day 4 p.i. was similar amongst all CD4^+^ T-cell subpopulations when compared to the RSV/CO control mice (Table S2). By day 7 p.i., the absolute cell counts and percent change of all CD4^+^ T cells in the RSV/PT2385 were of comparable number to the RSV/CO control mice. For CD8^+^ T-cell subpopulations, we observed a significant increase in the absolute number of CD8^+^ naïve cells at day 4 p.i., while the absolute cell counts for effector and active CD8^+^ T cells remained similar to the RSV/CO control mice (Figure 11E). At day 7 p.i., the absolute number of active CD8^+^ T cells was significantly increased, while the naïve and effector CD8^+^ cells remained comparable to the RSV/CO control mice (Figure 11E). The percent change was not modified for any CD8^+^ cells at either timepoint (Table S2). Collectively, these data show that the inhibition of HIF-2α results in increased T-cell numbers, which could contribute to better control of RSV replication, resulting in decreased viral titers.

**Figure 12:**
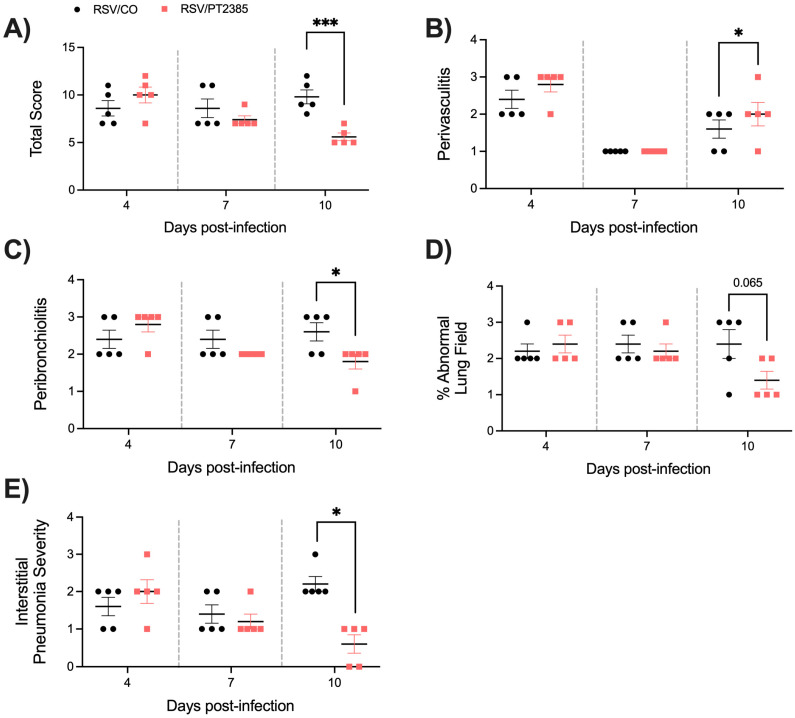
Histopathological scoring of lung tissue following HIF-2α inhibition during RSV infection. At days 4, 7, and 10 p.i., the left lung was collected from RSV-infected mice that had received CO or PT2385 and subjected to FFPE. Cuts of lung tissue were stained with H&E and observed under a microscope at 10× magnification. The (**A**) total score was calculated, consisting of scores for (**B**) perivasculitis, (**C**) peribronchiolitis, (**D**) percent abnormal lung field, and (**E**) interstitial pneumonia. Data are representative of one independent experiment (n = 5 mice/group). Data are expressed as mean ± SEM. Significant results were determined by an unpaired Student’s *t*-test (* *p* ≤ 0.05, *** *p* ≤ 0.001).

**Figure 13:**
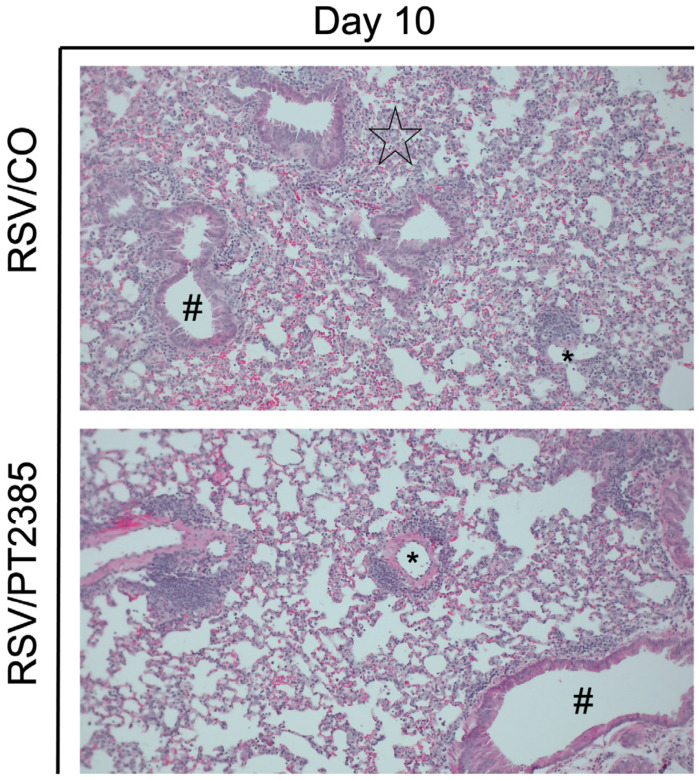
Histopathological images of lung tissue following HIF-2α inhibition during RSV infection. At day 10 p.i., the left lung was collected from mice that had received PBS or PT2385 and subjected to FFPE. Cuts of lung tissue were stained with H&E and observed under a microscope at 10× magnification. Perivasculitis is indicated by an asterisk. Peribronchiolitis is indicated by the hash symbol. Interstitial pneumonia is indicated by the black star. No interstitial pneumonia was present in RSV/PT2385. Representative images are shown here (n = 5 mice/group).

Lastly, we assessed the lung tissue of mice that received PT2385 by histopathology. Uninfected mice that received PT2385 remained comparable to the PBS/CO control mice in all categories and at all timepoints tested. RSV-infected mice treated with PT2385 demonstrated significant improvements at day 10 p.i. in total lung score (Figure 12A), peribronchiolitis (Figure 12C), and the severity of interstitial pneumonia (Figure 12E) as compared to the RSV/CO control mice. The percent abnormal lung field was found to have a trend towards improvement in RSV/PT2385 mice (Figure 12D). Additionally, there was a mild worsening of perivasculitis compared to the RSV/CO control mice (Figure 12B). Representative images of the lung tissue for RSV/CO and RSV/PT2385 mice are shown in Figure 13. Collectively, these data suggest HIF-2α does not modify airway function but does contribute to worsening lung pathology during the acute phase of RSV infection.

## 3. Discussion

The pharmacological use of HIF therapeutics has been extensively explored in various models of cancer, autoimmunity, and non-infectious lung damage [7,20,21,22]. While some studies have demonstrated the antiviral potential of HIF inhibitors against respiratory viruses, these findings have been primarily limited to in vitro characterizations [8]. Our laboratory has previously shown that RSV infection results in the stabilization of both HIF-1α and HIF-2α. Through the pharmacological inhibition of HIF, we also demonstrate the stabilization of HIF-1α but not HIF-2α to orchestrate metabolic reprogramming towards glycolysis and for this activity to be important for RSV replication in lung epithelial cells [14]. Given the diverse biological roles of HIF across various cellular compartments, we next sought to understand the effects of these inhibitors in a mouse model of RSV infection. In this study, we aimed to characterize the disease-modulating properties and antiviral potential of HIF-1α and -2α inhibitors in a BALB/c mouse model of RSV infection.

Our results show that the inhibition of HIF-1α worsens clinical disease while concurrently improving aspects of airway function and lung inflammation during RSV infection. In our initial experiments with PX478, when administering the inhibitor every day (QD), we observed notable weight loss and increased illness in the PBS/PXQD mice compared to the PBS control mice. As there was no difference in any other parameter analyzed, including BAL cells, this result suggests that the deteriorating clinical condition in PBS/PXQD mice is likely influenced by a factor other than inflammation or tissue damage. Previous studies investigating the anti-tumor efficacy of PX478 have administered this compound for up to 5 consecutive days, reporting minor weight loss in the mice during that time [15,21]. Studies using obese mouse models have noted PX478 to prevent weight gain [22,23], and a recent investigation has shown the efficacy of PX478 as a potential therapeutic for type II diabetes [24]. Considering the significant role of HIF-1α in metabolic functions and the recent repositioning of PX478 for the treatment of type II diabetes, it is possible that consecutive dosing of PX478 over extended periods of time may lead to appetite suppression. In our model, spacing treatments over 48 h (QAD) ameliorated this effect, resulting in patterns of clinical disease in the PBS/PXQAD mice that resemble previously reported observations. Despite this, RSV-infected mice treated with PX478 every 48 h continued to display delayed recovery as compared to the RSV control mice (Figure 1F). This was surprising, as these RSV-infected mice effectively cleared the virus from the lung tissue and showed no signs of increased inflammatory activity. One possible explanation for the phenomenon could be moderate appetite suppression induced by PX478, which is amplified due to the stress of infection.

Treatment with PX478 in RSV-infected mice resulted in initial antiviral activity regardless of the PX478 dosing schedule. This was followed by prolonged viral replication at later timepoints of infection when PX478 was given QD but not QAD. Concurrently, these mice experienced a significant reduction in the number of total cells present in the BAL, more strikingly in the number of lymphocytes. HIF-1α is critical for the activation, migration, and cytotoxic functions of T cells [25]. T cells play an important role in controlling RSV replication and clearance within the host [17,18,19]. During the early stages of RSV disease, CD4^+^ effector cells are known to aid in the recruitment and activation of other immune cells that control RSV replication, such as natural killer (NK) cells, CD8^+^ cytotoxic T cells, and B cells [26,27,28]. At later stages of disease, CD8^+^ effector T cells are responsible for directly clearing RSV from the lung epithelium [29]. RSV/PXQAD mice had a significant reduction in total leukocytes, total CD4^+^ T cells, and total CD8^+^ T cells at both timepoints assessed. Furthermore, during the peak of viral replication, the absolute number and percentage of active and effector CD4^+^ T cells were significantly reduced. Similarly, PXQAD treatment resulted in a significantly reduced number of active and effector CD8^+^ T cells present during peak viral clearance in the lungs of infected mice. Despite the overall impact on the T-cell population, the RSV/PXQD- and RSV/PXQAD-treated mice displayed antiviral activity at early and/or peak timepoints of RSV replication. Given the suppressed T-cell activity, this antiviral effect is more likely attributed to impaired RSV replication rather than immune-mediated activity. Consistent with our previous in vitro findings, the current study shows that HIF-1α inhibition in RSV-infected mice influences the expression of genes regulating critical metabolic pathways, including glycolysis, an essential pathway for RSV replication in airway epithelial cells [15]. This likely contributes to the reduced RSV replication at early and peak timepoints in mice rather than being a result of immune-mediated activity.

Several studies have shown CD8^+^ T cells to be responsible for the clearance of RSV from the lung tissue [29]. In our PXQD model, RSV replication is prolonged, indicating that the amount of virus that can replicate is not being cleared from the lung tissue (Figure S1). CD8^+^ T cells were found to be further suppressed in the RSV/PXQD mice when compared to the RSV/PXQAD mice. These results would suggest continuous treatment with PX478 to result in a threshold effect of CD8^+^ T-cell suppression, preventing efficient viral clearance. This is an important finding, considering the use of HIF-modulating therapeutics in the context of other diseases, such as cancer.

In contrast to HIF-1α, the inhibition of HIF-2α by PT2385 was found to improve clinical disease, with no difference in airway function. Like PX478, treatment with PT2385 demonstrated significant antiviral activity at early and peak timepoints of RSV replication, with no impact on viral clearance. Based on our previous characterizations in epithelial cells, where inhibition of HIF-2α did not affect metabolic reprogramming and viral replication [15], the mechanisms underlying this activity may be distinct from those responsible for impaired replication following HIF-1α inhibition.

RSV-infected mice treated with PT2385 displayed a moderate increase in immune cell presence in the airway space. This was characterized by an increased number of BAL macrophages and lymphocytes, as well as lung neutrophils and activated CD4^+^ and CD8^+^ T cells. Neutrophilic responses have been shown to contribute to the control of viral replication in several models of respiratory virus infection, including RSV, influenza, and SARS-CoV-2 [30,31,32,33], although other unidentified mechanisms are likely to play an important role in the early antiviral activity observed with PT2385 administration. Recent studies found that a loss of HIF-2α in regulatory T cells (Tregs) was associated with reduced suppressor functions, leading to an increase in CD4^+^ T-cell activity without changes in Treg development [34,35]. In agreement with this finding, our results indicate an increase in the number of CD4^+^ and CD8^+^ T cells, including active/effector cells, with no significant alteration in Treg cell numbers compared to the RSV/PBS control mice. As for other models of infection/inflammation, the loss of HIF protein activation in myeloid cells in a model of Leishmania infection also resulted in a heightened CD4^+^ Th1 immune response [36], and mice with a T-cell intrinsic HIF-2α deletion showed exacerbated intestinal inflammation in a model of experimental colitis [37]. Since HIF-2α was shown to be dispensable for RSV replication in epithelial cells, it is possible that the increased immune cell responses, both innate and adaptive, play a major role in the observed antiviral effect in RSV/PT2385 mice.

HIF-1α activation has been demonstrated to impact airway function in a variety of ways, including the induction of bronchoconstriction [38]. HIF-1α induces bronchoconstriction by influencing the contractility of airway smooth muscles through several mechanisms, including the modulation of calcium signaling, the regulation of the RhoA pathway, and the induction of inflammatory mediators [39,40,41]. In contrast, HIF-2α has been shown to be dispensable for the induction of changes to airway hypersensitivity and bronchoconstriction [34]. Consistent with these findings, we observed notable improvements in bronchoconstriction in RSV-infected mice treated with PX478 but not with PT2385. In the PXQAD experiments, pathology scores for perivasculitis and peribronchiolitis were significantly improved on days 4 and 7 p.i. as compared to the RSV control mice. This finding is consistent with the reduced immune cell infiltration and antiviral activity at these timepoints. As HIF-1α has important roles at the later stages of lung injury in promoting angiogenesis and tissue regeneration [42], it is possible that the inhibition of HIF-1α at the later stages of disease may hinder proper tissue repair, negating the benefit observed in the initial phase of RSV lung disease. Mice treated with PT2385 during RSV infection had no major alterations to any scored histopathology category until day 10 p.i. despite the observed increase in BAL cellularity and increased T-cell recruitment to the lung, which could possibly explain the lack of improvement in the airway function of these mice. By the end of the acute phase, RSV/PT2385 mice had a mild increase in perivasculitis but significant improvements in all other categories. This increase in perivasculitis could be due to the increase in the number of immune cells observed within the BAL and lung tissue but could also be related to changes in vascular function, as HIF-2α plays an important role in maintaining vascular integrity [43].

With the growing appreciation for the viral manipulation of the HIF pathway and the targeted therapeutic potential, several studies have begun to investigate the contribution of HIF proteins to diseases associated with other respiratory viruses. SARS-CoV-2 infection was found to induce the stabilization of HIF-1α in immune cells via mitochondrial damage, facilitating viral replication and cytokine production [44]. On the other hand, HIF activation via the prolyl hydroxylase inhibitor Roxadustat reduced angiotensin-converting enzyme 2 (ACE2) expression and inhibited SARS-CoV-2 entry and replication, both in lung epithelial cells [45] as well as in a hamster model of infection [46]. In vitro, IAV infection induces HIF stabilization via the inhibition of the proteasome and the decreased expression of factor-inhibiting HIF (FIH), with no changes in HIF-1α mRNA transcription or hydroxylation [47] and with the pharmacological inhibition of HIF significantly reducing viral replication [48]. In contrast, HIF-1α deficiency in the lung epithelium was found to facilitate viral replication in a mouse model of influenza A virus infection by promoting autophagy [49].

In conclusion, our data demonstrate that HIF-1α and HIF-2α are important regulators of the immune response to RSV infection. We also highlight HIF-1α as a critical component to the onset of RSV-induced bronchoconstriction. Additionally, these data describe the novel and complex contributions of the HIF-α subunits to RSV replication and clearance. Though we show antiviral potential for both HIF-α inhibitors, the resulting impact on clinical disease and the modulation of the immune responses warrants great care be taken when considering HIF-α inhibitors for therapeutic use against a viral respiratory infection. Furthermore, our data demonstrate that the continued inhibition of HIF-1α can result in prolonged viral replication, highlighting the potential unwanted side effects associated with the use of HIF-modulating therapeutics in the context of other diseases, such as cancer. A limitation of this study is the use of the rodent model, which is well established as an acceptable model of RSV infection but does not fully replicate human disease [50]. Additionally, our findings indicate the specificity of the inhibitors for their respective HIF targets, but the potential off-target effects of these inhibitors were not investigated. Future investigations will focus on further understanding the mechanistic contribution of the different HIF-α subunits to the immune response and tissue repair processes during RSV infection.

## 4. Materials and Methods

### 4.1. Animal Experiments

Female, 8- to 10-week-old BALB/c mice were purchased from Envigo (Indianapolis, IN, USA). Mice received either PX478 or PT2385 (HY-10231 and HY-12867, MedChemExpress, Monmouth Junction, NJ, USA) by oral gavage at a dose of 20 or 50 mg/kg, respectively. PX478 was dissolved in PBS, while PT2385 was dissolved in 10% DMSO + 90% Corn Oil (CO). For anti-HIF1α, PX478-treated mice were separated into three groups. The first received PBS as the control group. The second received PX478 every 24 h for 10 days (PXQD). The third group received PX478 every 48 h for 10 days (PXQAD). For anti-HIF2α, mice received either CO or PT2385 every 24 h for 10 days. Within 15 min of the first treatment, mice were anesthetized and intranasally (IN) inoculated with 50 mL of either PBS or RSV Long Strain at a dose of 5 × 10^6^ plaque-forming units (PFU). The preparation of RSV Long Strain (ATCC, Manassas, VA, USA) was performed as described previously [51]. All mice were monitored over the 10-day infection period for changes in body weight loss and clinical illness score on a 0-to-5 grading scale (0 = healthy, 1 = barely ruffled fur, 2 = ruffled fur but active, 3 = ruffled fur and inactive, 4 = ruffled fur, inactive and hunched, 5 = dead). All care and procedures involving mice in this study were completed in accordance with the recommendations in the Guide for the Care and Use of Laboratory Animals of the National Institutes of Health and the UTMB institutional guidelines for animal care. The Institutional Animal Care and Use Committee (IACUC) of UTMB approved these animal studies under protocol 9001002.

### 4.2. RNA Extraction and Reverse Transcription–Quantitative PCR (RT-qPCR)

Lung tissue was homogenized in TRIzol reagent ( Thermo Fisher Scientific, Waltham, MA, USA), and the RNA was isolated using a combination of the TRIzol-based method and the RNeasy Mini Kit (Qiagen, Hilden, Germany). Briefly, after phase separation with chloroform, the top aqueous layer was further processed using the Qiagen RNeasy Mini kit spin columns by following the manufacturer’s protocol. On-column DNase digestion was performed with the RNase-free DNase set (Qiagen). cDNA synthesis was performed with 1 µg of RNA using iScript Reverse Transcription Supermix (Bio-Rad Laboratories, Hercules, CA, USA). The cDNA was diluted four times with nuclease-free water, and the qPCR was performed using 4 µL of cDNA, pre-mixed probe and primers (TaqMan Gene Expression Assays, Applied Biosystems, Waltham, MA, USA), and TaqMan Universal Master Mix (Applied Biosystems). The Custom Plus TaqMan RNA Assay (Applied Biosystems) was used to assess the expression of RSV N (Assay ID: ARU66XH). The following mouse TaqMan Gene Expression Assays (4331182, Applied Biosystems) were used: Hif1*α* (Mm00468869_m1), Pgk1 (Mm00435617_m1), Hk2 (Mm00443386_m1), Slc2a1 (Mm00441480_m1), Serpine1 (Mm00435858_m1), Epo (Mm01202755_m1), and Epas1 (Mm01236112_m1). The Eukaryotic 18S rRNA Endogenous Control (Applied Biosystems) was used for normalization. The qPCR assays were run in the Bio-Rad CFX Connect real-time system. The delta-delta Ct method was used to calculate the relative changes in gene expression.

### 4.3. Bronchoalveolar Lavage and Viral Replication

The BAL fluid was obtained as previously described [16]. A small aliquot was used to determine the total cell count and cellular differentiation of the BAL by cytospin analysis. The remaining BAL fluid was centrifuged, and the supernatants were collected and stored at −80 °C. To assess viral replication, lungs were collected from mice at days 2, 4, 7, and 10 post-infection (p.i.) to perform plaque assays, as previously described [16].

### 4.4. Flow Cytometry

At days 1, 2, 4, and 7 p.i., mice were euthanized, and the whole lung was collected. The tissue was minced and digested with 10 mg/mL DNase I (Sigma-Aldrich, St. Louis, MO, USA) and 50 mg/mL collagenase IV (Worthington Biochemical, Lakewood, NJ, USA). The tissue was incubated at 37 °C for 30 min and then passed through a 70 μm cell strainer in RPMI 1640 medium + 10% FBS (CRPMI), and red blood cells were removed by using Red Blood Cell Lysis Buffer (Sigma-Aldrich, Burlington, MA, USA). TruStain FcX (Biolegend, San Diego, CA, USA) was first used to reduce non-specific binding, followed by Zombie NIR Viability dye. To assess cellular populations of neutrophils, alveolar macrophages, and natural killer (NK) cells at days 1 and 2 p.i., a comprehensive panel of fluorochrome-labeled antibodies was utilized. The antibodies employed included FITC-anti-CD45 (clone 30-F11), BV785-anti-CD11b (clone M1/70), PerCP/Cyanine 5.5-anti-Ly6G (clone 1A8), BUV395-anti-CD11c (clone HL3), PE-Cy7-anti-F4/80 (clone BM8), BV510-anti-MHCII (clone M5/114.15.2), BV711-anti-CD3ε (clone 145-2C11), and PE/Dazzle 594-anti-CD49b (clone DX5). For the analysis of CD4^+^ and CD8^+^ T-cell populations at days 4 and 7 p.i., the panel included FITC-anti-CD45 (clone 30-F11), BV785-anti-CD3 (clone 17A2), AF 700-anti-CD4 (clone GK1.5), BV510-anti-CD8a (clone 53-6.7), PerCP/Cy5.5-anti-CD44 (clone IM7), BV605-anti-CD62L (clone MEL-14), and PE/Cy7-anti-CD69 (clone H1.2F3). All antibodies were purchased from Biolegend, Thermo Fisher Scientific, Novus Biologicals, or R&D Systems and titrated prior to use. Data were acquired on a BD FACSymphony A5 SE in the UTMB Flow Cytometry and Cell Sorting Core and analyzed using FlowJo software version 10.9.0 (BD Bioscience, Franklin Lakes, NJ, USA).

### 4.5. Assessment of Airway Function and Lung Histopathology

Bronchoconstriction was evaluated by measuring Penh values in unrestrained mice using whole-body barometric plethysmography (Buxco, Troy, NY, USA) as previously described [52]. Total protein was measured using BioRad Total Protein Dye (Bio-Rad Laboratories, Hercules, CA, USA). For histology, the left lung was collected from mice at days 4, 7, and 10. The tissue was fixed in 10% neutral buffered formalin and subjected to paraffin embedding. Sections were cut and stained with H&E and evaluated by a board-certified pathologist with expertise in mouse lungs (Y.L.J.-H). The assessment of the tissue was performed as previously described [53].

### 4.6. Statistical Analysis

Statistical analyses were performed using a two-way mixed ANOVA or an unpaired Student’s *t*-test as appropriate. Analyses were performed using GraphPad Prism 9.5.1 (GraphPad Software, Inc., San Diego, CA, USA). Results are expressed as mean ± SEM, and *p* ≤ 0.05 value was selected to indicate significance.

## Data Availability

All data generated and analyzed to support the findings of this study are included within the article and its additional files.

## Supplementary Materials

The following supporting information can be downloaded at: https://www.mdpi.com/article/10.3390/ijms26073182/s1.
