# Supplementary material for: Role of Hypoxia-Inducible Factors in Respiratory Syncytial Virus Infection-Associated Lung Disease
Source: Int J Mol Sci. 2025 Mar 29;26(7):3182. doi: 10.3390/ijms26073182 (PMC11989216; doi:10.3390/ijms26073182)
Supplement: Supplementary file 1 [file ijms-26-03182-s001.zip › ijms-3478619-supplementary.pdf]

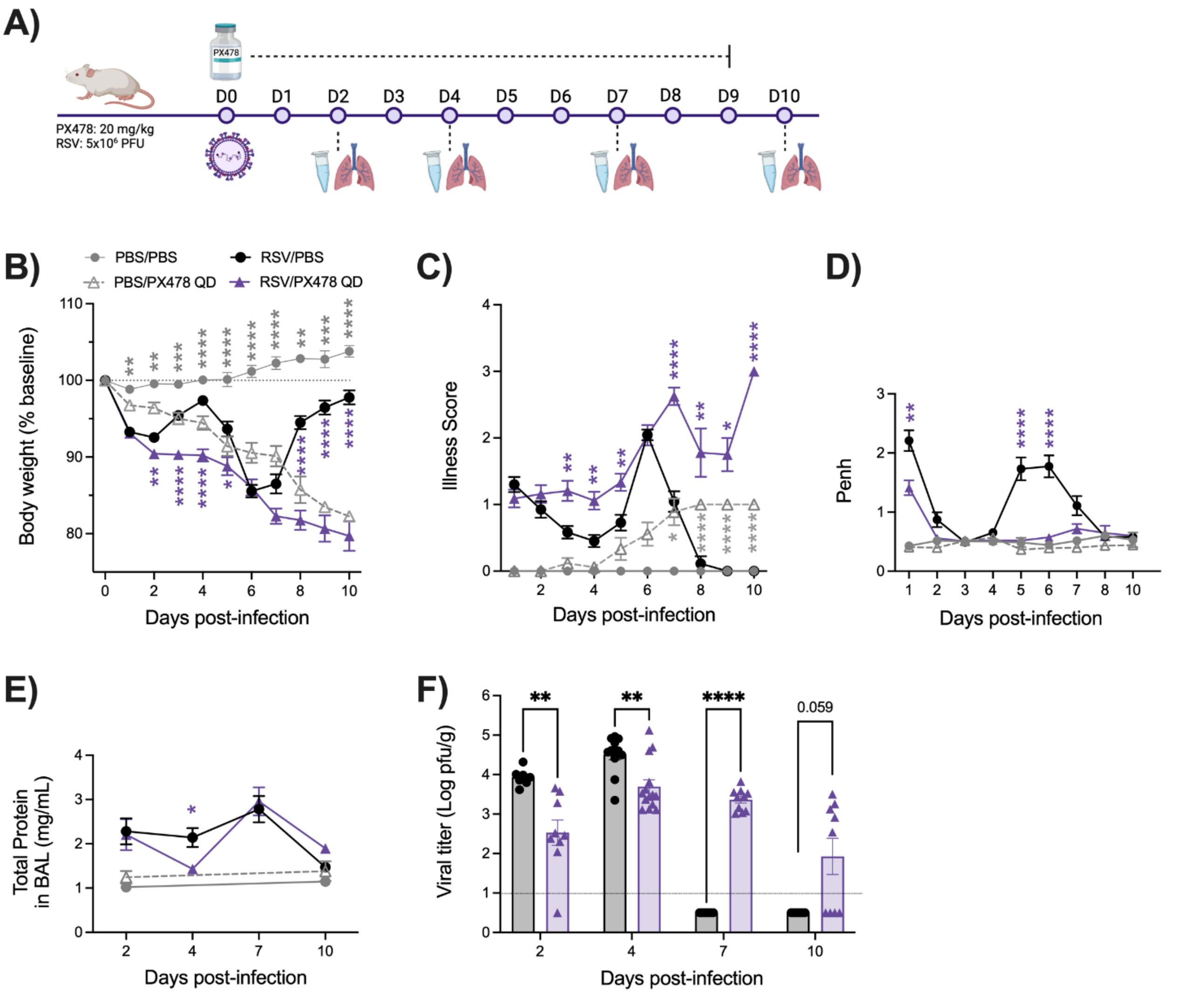

Figure S1. *Assessment of clinical disease, airway function, and viral replication following HIF-1a inhibition during RSV infection.* The experimental design for mice treated with PX478 QD is described in (A). Following treatment, all mice were monitored daily for changes in (B) body weight and (C) illness score. (D) Bronchoconstriction, represented by baseline Penh, was measured by plethysmography. At days 2, 4, 7, and 10 p.i., (E) total protein was measured in the bronchoalveolar lavage (BAL) fluid and the right lung was collected for assessment of (F) viral replication by plaque assay. For clinical disease, data are pooled from four independent experiments (PBS/PBS n = 6, PBS/PX n = 6-18, RSV/PBS and RSV/PX n = 6-24 mice/group). For bronchoconstriction, data are pooled from three independent experiments (PBS/PBS and PBS/PX n = 6, RSV/PBS and RSV/PX n = 6-24 mice/group). For total protein, data are pooled from two independent experiments (PBS/PBS and PBS/PX n = 4, RSV/PBS and RSV/PX n = 8-10 mice/group). For viral replication, data are pooled from two independent experiments (RSV/PBS n = 7-12, RSV/PX n = 9-14 mice/group). Data are expressed as mean ± SEM. Significant results were determined by two-way mixed ANOVA (* $p \leq 0.05$, ** $p \leq 0.01$, *** $p \leq 0.001$, **** $p \leq 0.0001$).

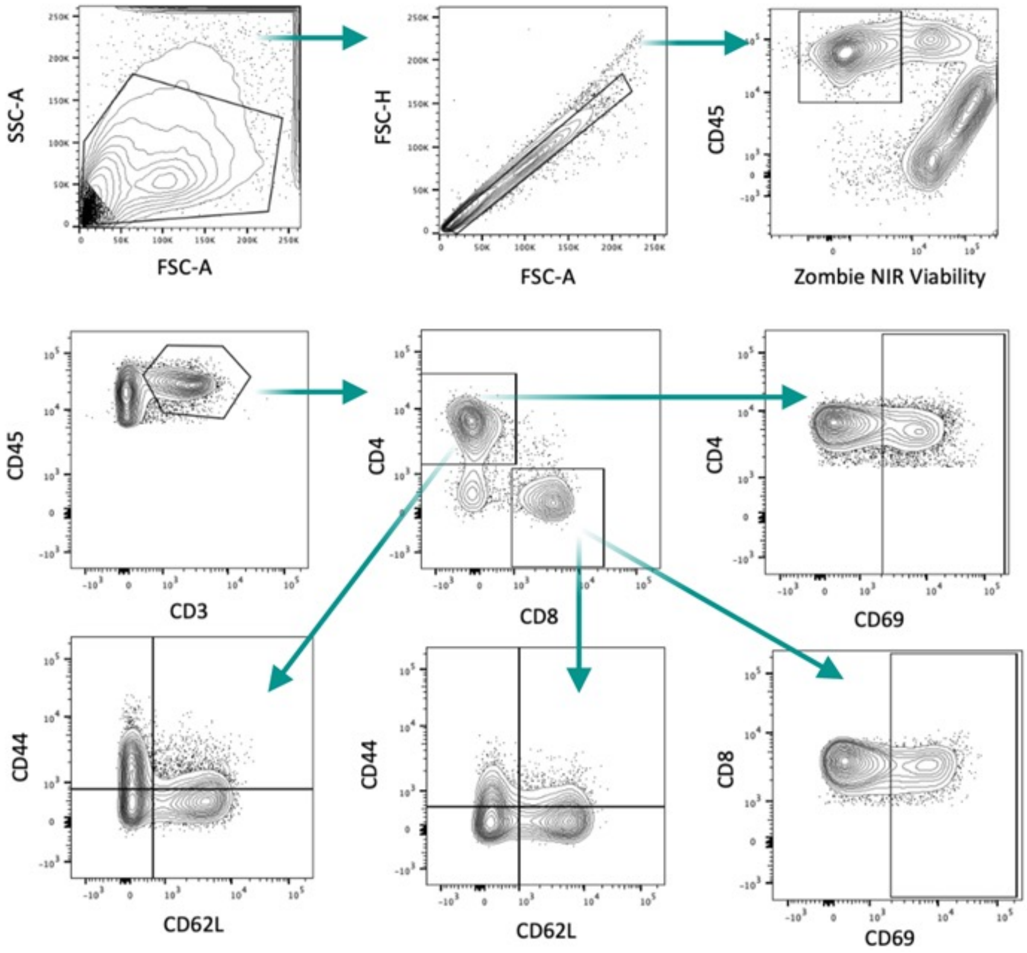

Figure S2. *Schematic for analysis of CD4$^+$ and CD8$^+$ T-cells by flow cytometry.* At days 4 and 7 p.i., whole lung tissue was collected from the respective groups. Single cell suspension was prepared, stained with live/dead cell dye and fluorochrome-conjugated antibodies, followed by flow cytometric analysis. T cells were gated on CD45$^+$CD3$^+$ first, then further gated on CD4$^+$ and CD8$^+$ subpopulations. Naïve and effector T cells were gated on CD44$^-$CD62L$^+$ and CD44$^+$CD62L$^-$, respectively. CD69 was used as an early activation marker of T cells.

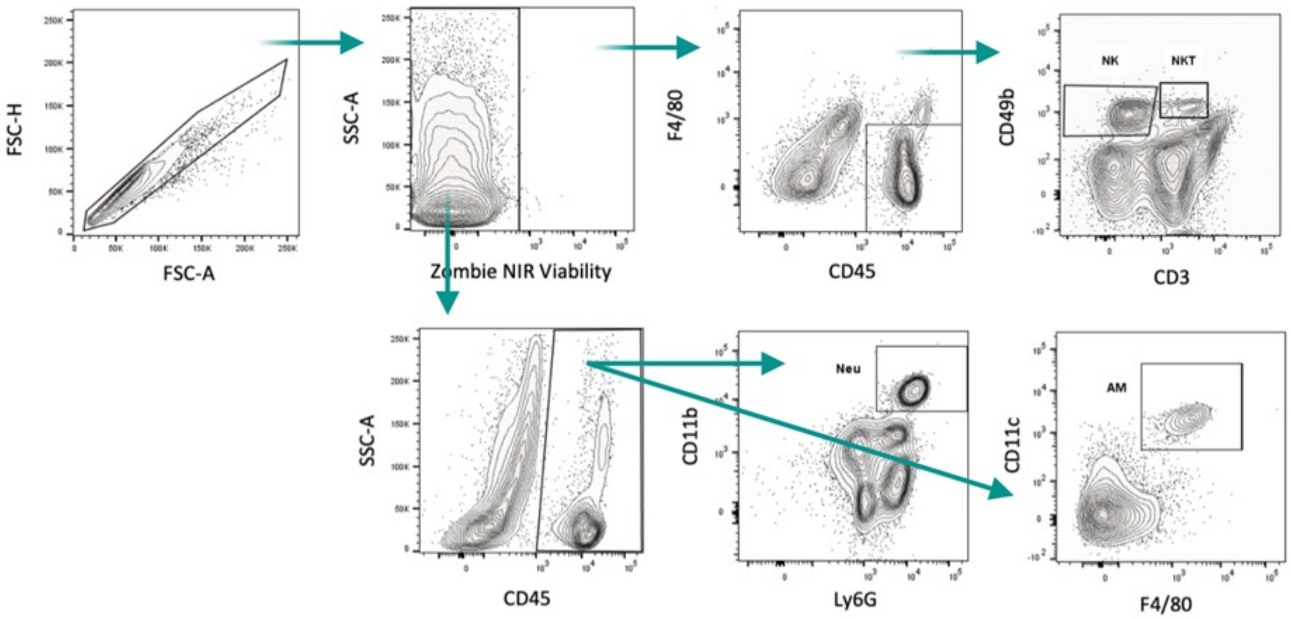

<u>Figure S3.</u> *Schematic for analysis of neutrophils, alveolar macrophages (AM), natural killer (NK), and NKT cells by flow cytometry.* At days 1 and 2 p.i., whole lung tissue was collected from the respective groups. Single cell suspension was prepared, stained with live/dead cell dye and fluorochrome-conjugated antibodies, followed by flow cytometric analysis. After gating for $Live^{+}/Dead^{-}$ followed by $F4/80^{-}CD45^{+}$ leukocytes, NK cells were gated on $CD3^{-}CD49^{+}$, and NKT cells were gated on $CD3^{+}CD49^{+}$. After gating for $Live^{+}/Dead^{-}$ followed by $CD45^{+}$ leukocytes, neutrophils were gated on $CD11b^{+}Ly6G^{+}$ and AMs were gated on $CD11c^{+}F4/80^{+}$.

Table S1. Percentages of $CD4^+$ and $CD8^+$ T cells subpopulations following HIF-1α inhibition at days 4 and 7 post RSV infection. Data from two independent experiments (n = 9-10 mice/group).

	RSV/PBS						RSV/PX478 QAD					
	Percentage from $CD4^+$			Percentage from $CD8^+$			Percentage from $CD4^+$			Percentage from $CD8^+$		
	Active	Eff	Naive	Active	Eff	Naive	Active	Eff	Naive	Active	Eff	Naive
**D4**	47.3	34.9	38.1	30.4	14.1	49.2	29.4	27.3	49.5	17.1	8.1	59.9
**D7**	16.8	37.7	22.5	58.8	65.2	10.0	18.7	37.1	20.9	62.9	61.0	10.6

Table S2. Percentages of $CD4^+$ and $CD8^+$ T cells subpopulations following HIF-2α inhibition at days 4 and 7 post RSV infection. Data from two to three independent experiments (n = 10-14 mice/group).

	RSV/CO						RSV/PT2385					
	Percentage from $CD4^+$			Percentage from $CD8^+$			Percentage from $CD4^+$			Percentage from $CD8^+$		
	Active	Eff	Naive	Active	Eff	Naive	Active	Eff	Naive	Active	Eff	Naive
**D4**	11.0	23.1	45.8	17.8	17.8	45.1	10.4	24.4	46.0	17.7	19.4	43.9
**D7**	19.3	45.6	22.3	47.6	54.4	14.0	19.0	44.8	20.5	49.7	53.4	12.3
